# CPEB1 orchestrates a fine-tuning of miR-145-5p tumor-suppressive activity on TWIST1 translation in prostate cancer cells

**DOI:** 10.18632/oncotarget.27806

**Published:** 2020-11-10

**Authors:** Fatemeh Rajabi, Win-Yan Liu-Bordes, Marina Pinskaya, Foretek Dominika, Gueorgui Kratassiouk, Guillaume Pinna, Simona Nanni, Antonella Farsetti, Christian Gespach, Arturo Londoño-Vallejo, Irina Groisman

**Affiliations:** ^1^Telomeres and Cancer Laboratory, CNRS, Sorbonne Université, Université PSL, Institut Curie, Paris, France; ^2^Non-Coding RNA, Epigenetic and Genome Fluidity, Sorbonne Université, Université PSL, Institut Curie, Paris, France; ^3^Plateforme ARN Interférence, Service de Biologie Intégrative et de Génétique Moléculaire (SBIGeM), Gif-sur-Yvette, France; ^4^Istituto di Biologia Cellulare e Neurobiologia, Consiglio Nazionale delle Ricerche (CNR), Rome, Italy; ^5^Istituto di Patologia Medica, Università Cattolica del Sacro Cuore, Rome, Italy; ^6^Fondazione Policlinico Universitario A. Gemelli IRCCS, Roma, Italy; ^7^Sorbonne Université, Inserm U938, Team TGFβ Signaling in Cellular Plasticity and Cancer, Centre de Recherche Saint-Antoine, Paris, France

**Keywords:** miR-145-5p, TWIST1, CPEB1, EMT, prostate cancer

## Abstract

TWIST1 is a basic helix-loop-helix transcription factor, and one of the master Epithelial-to-Mesenchymal Transition (EMT) regulators. We show that tumor suppressor miR-145-5p controls TWIST1 expression in an immortalized prostate epithelial cell line and in a tumorigenic prostate cancer-derived cell line. Indeed, shRNA-mediated miR-145-5p silencing enhanced TWIST1 expression and induced EMT-associated malignant properties in these cells. However, we discovered that the translational inhibitory effect of miR-145-5p on TWIST1 is lost in 22Rv1, another prostate cancer cell line that intrinsically expresses high levels of the CPEB1 cytoplasmic polyadenylation element binding protein. This translational regulator typically reduces TWIST1 translation efficiency by shortening the TWIST1 mRNA polyA tail. However, our results indicate that the presence of CPEB1 also interferes with the binding of miR-145-5p to the TWIST1 mRNA 3′UTR. Mechanistically, CPEB1 binding to its first cognate site either directly hampers the access to the miR-145-5p response element or redirects the cleavage/polyadenylation machinery to an intermediate polyadenylation site, resulting in the elimination of the miR-145-5p binding site. Taken together, our data support the notion that the tumor suppressive activity of miR-145-5p on TWIST1 translation, consequently on EMT, self-renewal, and migration, depends on the CPEB1 expression status of the cancer cell. A preliminary prospective study using clinical samples suggests that reconsidering the relative status of miR-145-5p/TWIST1 and CPEB1 in the tumors of prostate cancer patients may bear prognostic value.

## INTRODUCTION

Prostate cancer (PCa) is a leading cause of cancer-related deaths in men. Initial surgical or hormonal treatments are succeeded by a relatively high incidence of recurrence, often attributed to the persistence of stem cell-like cancer cells. Also, a significant fraction of prostate tumors displays initial aggressive behavior, often metastasize to bone, leading to significant mortality. Similar to what occurs in other carcinomas, Epithelial-to-Mesenchymal Transition (EMT), a process characterized by decreased expression of epithelial markers and increased mesenchymal markers, plays a critical role in prostate cancer cell invasion, metastasis, and tumor recurrence, by conferring cancer tumor cells with higher migration capacity and chemotherapy resistance [1–3].

The basic Helix-Loop-Helix (bHLH) transcription factor TWIST1 is one of the six transcription factors that control EMT during normal development, but that can also promote EMT and tumor metastasis in cancer cells [4, 5]. Indeed, overexpression of TWIST1 has been observed in various types of cancer, including breast, prostate, gastric, pancreatic, bladder, and hepatocellular carcinoma, as well as in rhabdomyosarcoma and glioma, and is often associated with more aggressive phenotypes and acquired drug resistance, as reviewed in [6]. An increase in TWIST1 expression has been documented with respect to prostate cancer progression, since its overexpression correlates with a high Gleason score [7]. TWIST1 expression is regulated by a complex signaling network and has been described as an integrator of SHH, FGF, and BMP-signaling [8]. During mammalian embryogenesis, *TWIST1* mRNA precedes TWIST1 protein expression, indicating translational control of TWIST1 [9]. The same phenomenon has been observed in MCF-10ANeoT cells undergoing EMT [10].

MicroRNAs have emerged as critical post-transcriptional negative regulators of EMT, one of which is miR-145-5p, whose down-regulation has been widely documented in PCa [11–16]. Moreover, down-regulation of miR-143 and miR-145-5p, which belong to the same cluster, is associated with the induction of EMT and PCa bone metastasis [17]. MiR-145-5p expression is controlled by DNA methylation and by the tumor suppressor p53, which are often boss lost in aggressive PCa [18, 19]. Experimentally, it has been shown that p53 up-regulates expression of miR-145-5p, thereby suppressing metastasis and EMT. This effect was reversed by miR-145-5p down-regulation in prostate cancer-derived PC3 cells (19). In addition to prostate cancer, miR-145-5p tumor suppressor activity has been suggested in a variety of tumors, including bladder, breast, colorectal, gastric, lung, oral, and ovarian carcinomas [20]

Very few miR-145-5p targets are known to be directly involved in PCa EMT and metastasis. Validated miR-145-5p targets include EMT transcription factor ZEB2 [21], and the cytoplasmic scaffolding protein and human enhancer of filamentation1 (HEF1), which is also known as NEDD9/Cas-L [22]. TWIST1 is another potential target of miR-145-5p that could be involved in PCa progression and treatment [7]. Mouse *TWIST1* 3′UTR bears regulatory sites predicted to bind miR-145-5p, among a few other miRNAs that operate during mouse early development [23].

Previous work has shown that cytoplasmic polyadenylation element binding protein (CPEB1), another post-transcriptional regulator of gene expression, interacts with and down-regulates *TWIST1* mRNA expression by controlling the length of its polyA tail [10, 24]. CPEB1-depleted mammary epithelial tumor cells alter their gene expression profile in a manner consistent with EMT, and become motile [25]. CPEB1 depletion has been associated with the capacity of malignant cells to promote invasion and angiogenesis [26, 27]. Of note, CPEB1 levels are decreased in several types of human tumors, including ovary, stomach and breast cancers, as well as in myeloma. In this work, we have discovered so-far unanticipated molecular interplay between miR-145-5p and CPEB1, two critical effectors involved in controlling TWIST1 translation and therefore in EMT, stem cell self-renewal, and their associated transforming functions. A complementary prospective study with clinical prostate cancer samples has suggested that miR-145-5p and/or CPEB1 deficiencies are associated with TWIST1-dependent promotion of tumor growth and metastasis.

## RESULTS

### The differential impact of MiR-145-5p on TWIST1 expression in human prostate epithelial cell lines is dependent on CPEB1

**Figure 1:**
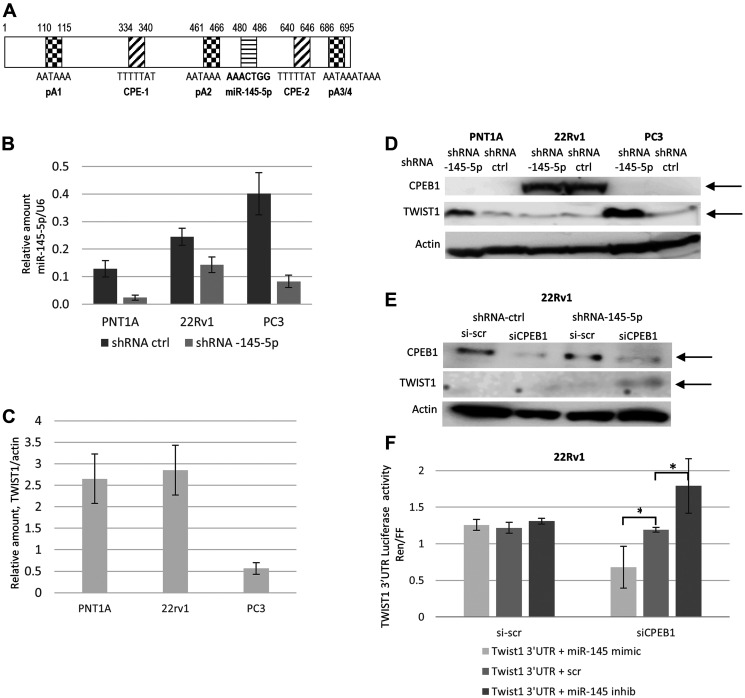
Interplay between miR-145-5p, CPEB1, and TWIST1 3′UTR regulation on TWIST1 expression in the PC3, 22Rv1 and PNT1A human prostate cell lines. (**A**) Schematic representation of *TWIST1* 3′UTR and its regulatory elements. Numbers correspond to the sequence of the following specified regulatory elements: pA1, pA2, and pA3/4 polyadenylation sequences (hexanucleotides), where shortening and polyadenylation of the 3′UTR takes place; the CPE-1 and CPE-2 cytoplasmic polyadenylation elements, and the miR-145-5p RE recognition site. (**B**) Lentivector-based anti-miR-145-5p ShRNA (MiRZIP-145) compared to miRZIP control vector down-regulation of miR-145-5p expression in PNT1A, PC3, and 22Rv1 cells, evaluated by RTqPCR. (**C**) RTqPCR analysis of *TWIST1* mRNA expression in PNT1A, 22Rv1, and PC3 cells. (**D**) Down-regulation of miR-145-5p expression by shRNA (miRZip Lentivector-based anti-microRNAs) up-regulates TWIST1 expression in PNT1A and PC3, but not in 22Rv1 cells characterized by high CPEB1 protein levels. (**E**) SiCPEB1 knockdown combined with sh-antagomir miR-145-5p increased TWIST1 expression in 22Rv1 cells by Western blotting. (**F**) Co-transfection of 22Rv1 cells of dual luciferase reporter constructs, in which Renilla luciferase attached to *TWIST1* 3′UTR-wt with miR-145 mimic decreased luciferase activity, whereas co-transfection with miR-145-5p antagomir increased reporter activity only in siCPEB1-silenced cells. Data are presented as the Renilla-to-Firefly luciferase activity ratio (Ren/FF). Experiments were repeated 3 times.

Our aim was to investigate whether TWIST1 expression is under post-transcriptional control in PCa cells. Analysis of the *TWIST1* 3′UTR sequence revealed a few elements that potentially influence the regulation of TWIST1 expression: an miR-145-5p response element (RE), two cytoplasmic polyadenylation elements (CPE), and three polyadenylation sites (PA) (Figure 1A).

We first tested the ability of miR-145-5p to regulate TWIST1 expression levels in immortalized and tumorigenic human prostate epithelial cells. For this purpose, we introduced a lentiviral vector expressing either an shRNA against miR-145-5p (miRZIP-145-5p) or a control shRNA (miRZIP-ctrl) into the PNT1A immortalized prostate epithelial cell line and, into the androgen-responsive 22Rv1 prostate cancer cell line, as well as into the androgen-independent PC3 prostate cancer cell line. In PNT1A and PC3 cells that expressed miRZIP-145-5p showed up to an 80% depletion of miR-145-5p compared to control cells, while only a 40% miR-145-5p depletion was observed in 22Rv1 cells that expressed miRZIP-145-5p (Figure 1B). With respect to TWIST1, control PNT1A and 22Rv1 cells contained four times more *TWIST1* mRNA compared to PC3 cells (Figure 1C). MiR-145-5p shRNA induced strong up-regulation of TWIST1 protein in both PC3 and PNT1A cells, but not in 22Rv1 cells (Figure 1D). Therefore, although depletion of miR-145-5p in 22Rv1 cells was limited, *TWIST1* mRNA and protein levels were not affected in this particular cell line, suggesting that miR-145-5p-dependent control of TWIST1 could depend on the cell type.

As noted above, along with the miR-145-5p response element, *TWIST1* 3‘UTR carries two CPE sites that have been shown to bind CPEB1, a protein also known to negatively control TWIST1 expression (Figure 1A). It has been proposed that CPEB1 works cooperatively with miR-580 to further down-regulate TWIST1 protein expression in breast epithelial cell line [10, 24]. Interestingly, we found that CPEB1 is robustly expressed in 22Rv1 cells, but not in PNT1A and PC3 cells (Figure 1D). To explore potential interplay between CPEB1, miR-145-5p, and *TWIST1* mRNA in 22Rv1 cells, we depleted CPEB1 up to 70%, using siRNAs in both miR-145-5p KD and control 22Rv1 cells (Figure 1E). Depleting CPEB1 by itself had no impact on TWIST1 protein levels in otherwise unperturbed 22Rv1 cells. However, 22Rv1 cells depleted in both miR-145-5p and CPEB1 showed increased TWIST1 protein levels (Figure 1E). Thus, miR-145-5p is able to negatively regulate TWIST1 only in the absence of CPEB1, thus providing an explanation for the cell-specific context of miR-145-5p activity against TWIST1. Furthermore, the proximity both of CPEB1 and miR-145-5p binding elements in the 3′UTR of *TWIST1* mRNA suggests that CPEB1 may physically interfere with miR-145-5p binding.

To confirm that the 3′UTR of *TWIST1 mRNA* was the target of CPEB1 that prevented miR-145-5p from acting as a repressor, we used a luciferase reporter construct comprising a reporter gene attached to *TWIST1* 3′UTR in 22Rv1 cells depleted or not in CPEB1 (Figure 1F). Transfection of 22Rv1 cells with either miR-145-5p mimic or antagomir respectively reduced or increased luciferase/TWIST1 expression exclusively in siCPEB1 depleted cells, not in cells with unperturbed CPEB1 levels. These results strengthen the hypothesis that the presence of CPEB1 determines whether or not miR-145-5p can act as inhibitor of TWIST1 expression.

**Figure 2:**
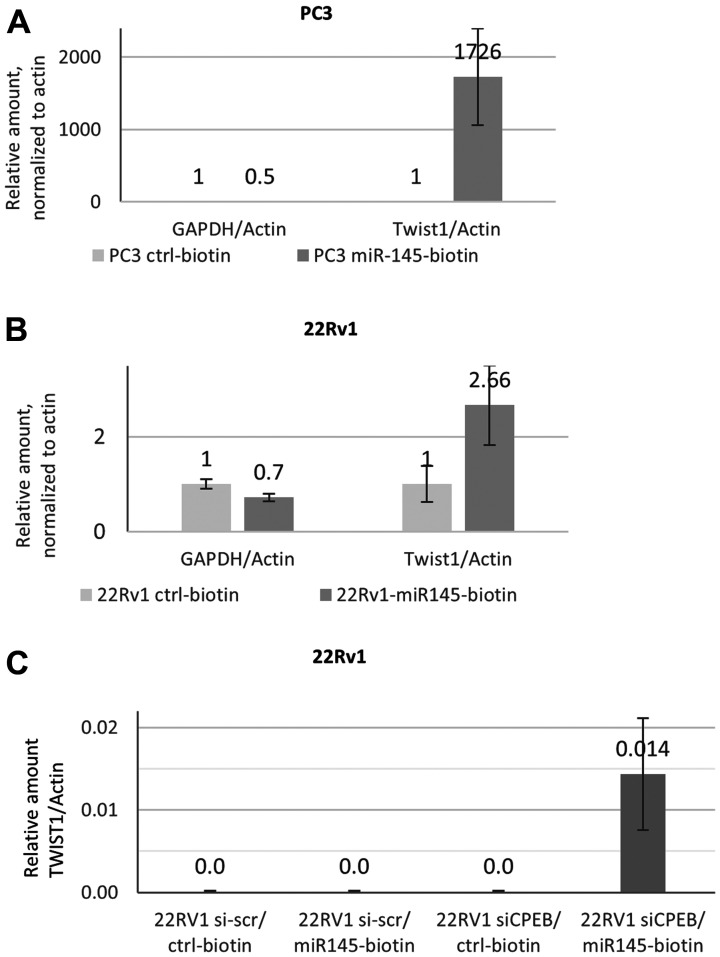
Mir-145-5p interaction with TWIST1 mRNA depends on CPEB1 in prostate cancer cell lines. (**A** and **B**) RTqPCR analyses of *TWIST1* mRNA co-precipitated with biotinylated miR-145 on streptavidin beads from prostate cancer cells revealed better accessibility of TWIST1 in PC3 (A) than in 22RV1 cells (B). GAPDH was used as the control for non-specific binding to streptavidin beads. Actin was used for RTqPCR normalization. (**C**) Biotinylated miR-145 precipitates *TWIST1* mRNA from the 22Rv1 cell were detected by qPCR after siCPEB silencing. Actin was used for RTqPCR normalization. Experiments were repeated twice.

To further confirm that miR-145-5p binding to *TWIST1* mRNA is compromised in 22Rv1 cells expressing CPEB1, we carried out pull-downs with biotinylated miR-145-5p from 22Rv1 cells extract and quantified the amount of miR-145-5p-associated *TWIST1* mRNA. As illustrated in Figure 2A and 2B, *TWIST1* mRNA was abundantly recovered in PC3 but not in 22Rv1 cells, even though the former had lower *TWIST1* mRNA levels (Figure 1C). To verify that CPEB1 expression is responsible for the failure of miR-145-5p to bind to *TWIST1* mRNA, we carried out biotinylated miR-145-5p pull-downs, using extracts from control and CPEB1-depleted 22Rv1 cells. As illustrated in Figure 2C, *TWIST1* mRNA could only be recovered from 22Rv1 cells in which CPEB1 had been knocked-down, providing strong support for the conclusion that CPEB1 does prevent the binding of miR-145-5p to *TWIST1* 3′UTR in 22Rv1 cells.

**Figure 3:**
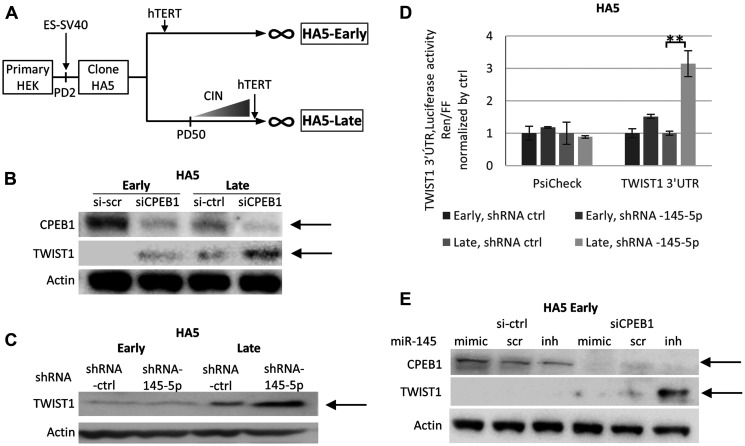
The CPEB1/Mir-145-5p interplay controls TWIST1 expression during telomere shortening in HEK cells. (**A**) Primary human embryonic kidney HEK cells were immortalized by ER-SV40 and hTERT, according to the production of karyotypically stable cells (at Early passages, < PD50 in HA5-Early cells, CIN-) and karyotypically unstable cells (at Late passages, >PD50 in HA5-Late cells, CIN+) [28]. (**B**) SiCPEB1 Knock-down (KD) in HA5 Early and Late cells led to up-regulation of TWIST1 expression detected by Western blot analysis. (**C**) Western blot analysis shows TWIST1 up-regulation induced by the shRNA of MiR-145-5p (MiRZIP-145-5p) KD, in HA5 Late cells but not Early cells. (**D**) MiR-145-5p shRNA KD increases the relative ratio of Renilla *versus* Firefly luciferase activity derived from the Renilla gene attached to *TWIST1* 3′UTR in Late cells but not in Early cells. The Renilla gene alone did not show any difference in luciferase expression. (**E**) MiR-145-5p mimic or antagomir introduced in Early cells controls TWIST1 expression only after siCPEB1 KD, as shown by Western blot analysis. Experiments were repeated three times.

To confirm the permissive role of CPEB1 on the activity of the miR-145-5p TWIST1 suppressor, we used a cell transformation model (Figure 3A) in which human epithelial kidney cells (HEK) undergo EMT in tight association with the accumulation of chromosomal instability (CIN+) due to telomere-shortening [28]. HEK cells (clone HA5) immortalized during early passages (Early cells), remained epithelial and displayed no CIN. On the other hand, Late cells displayed strong and stable EMT after initiation of CIN [28]. This EMT is associated with massive down-regulation of the miR-200 family and up-regulation of EMT transcription factors [28], including TWIST1, as shown in Figure 3B. Immunoblot analysis also revealed that Early cells displayed higher levels of CPEB1 compared to Late cells (Figure 3B). As expected, depletion of CPEB1 in both cell types led to increased TWIST1 expression (Figure 3B). However, we observed that, similar to the situation described above for prostate cancer cells 22Rv1, stable shRNA down-regulation of miR-145-5p led to an increase in TWIST1 expression in the context of low CPEB1 expression (Late cells), but not in the context of high CPEB1 expression (Early cells) (Figure 3C). To ascertain that the observed differences in miR-145-5p down-regulation on TWIST1 expression in Early and Late cells depended on *TWIST1* 3′UTR but not on other factors, we used the luciferase-reporter assay described above. As shown in Figure 3D, miR145-5p down-regulation led to an increase in *TWIST1* 3′UTR-dependent luciferase expression level in Late but not in Early cells. Furthermore, to verify that the lack of effect of miR-145-5p on TWIST1 expression in Early cells depended on CPEB1, we depleted CPEB1 in Early cells that expressed either a mimic or an inhibitor of miR-145-5p. We observed that in cells in which CPEB1 was depleted, the presence of miR-145-5p mimic led to down-regulation of TWIST1, whereas the presence of miR-145-5p inhibitor led to its up-regulation (Figure 3E). These results further corroborate the existence of CPEB1/miR-145-5p interplay with respect to the regulation of TWIST1 expression.

### Disentangling interplay between the miR-145-5p response element (RE) and cytoplasmic polyadenylation elements (CPEs) in TWIST1 3′UTR translation

**Figure 4:**
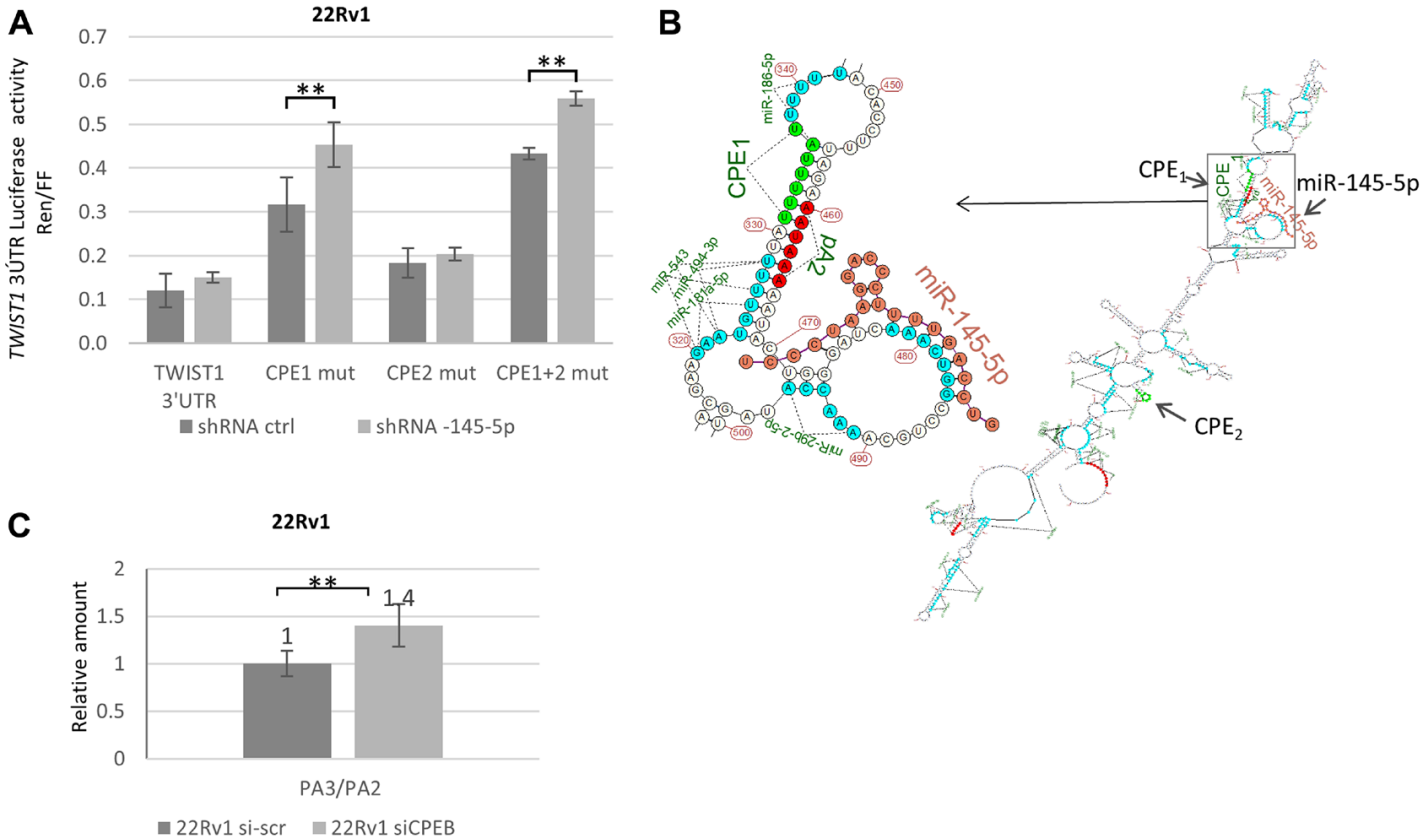
Molecular interactions between CPEB1 and miR-145-5p toward TWIST1 3′UTR are implicated in TWIST1 translation. (**A**) ShRNA-145-5p KD stimulates luciferase expression from the Renilla-luciferase (R-luc) gene attached to the *TWIST1* 3′UTR, with site-specific mutations in the CPE1 and CPE1+2 binding sites, but not in the CPE2 site alone. (**B**) Predicted secondary structure of *TWIST1* 3′UTR showing close proximity of CPE1 (but not CPE2) and miR-145-5p RE in *TWIST1* 3′UTR. *TWIST1* 3′UTR 2D-fold structure was generated with the help of the service http://unafold.rna.albany.edu/?q=mfold/RNA-Folding-Form. Folding results in XRNA ss format are rendered using custom script in R with miRNAs matching sites marked and hsa-mir-145-5p structure fold co-folded. (**C**) RTqPCR analysis of *TWIST1* 3′UTR with PA2 and PA3 primers reveal a 40% increase in the quantity of PA3 versus PA2 domains in *TWIST1* 3′UTR after siCPEB1 silencing. Si-scrambled was used as an irrelevant control. Experiments were repeated three times.

Since *TWIST1* 3′UTR bears two CPEB1 binding sites (CPE-1 and CPE-2) surrounding the miR-145-5p response element (RE) domain (Figure 1A), we sought to determine which site was important in preventing miR-145-5p binding. For this purpose, we used the same luciferase reporter assay and introduced mutations in either CPE-1, CPE-2, or both into the *TWIST1* 3′UTR [10]. The constructs were introduced into 22Rv1-transfected cells that expressed miRZIP-ctrl or miRZIP-145-5p. As shown in Figure 4A, these experiments revealed that CPE-1 is not only necessary but sufficient to allow CPEB1 to interfere with miR-145-5p activity against *TWIST1* 3′UTR. Indeed, abrogation of this particular CPE-1 site resulted in increased luciferase expression when miR-145-5p is down-regulated. Interestingly, if we consider the optimal predicted secondary structure of the *TWIST1* 3′UTR (obtained from the “Unafold.RNA” prediction browser), it appears that the miR-145-5p RE is positioned in close vicinity to CPE-1 but not to CPE-2. This observation reinforces the idea that CPEB1 could physically hinder miR-145-5p binding to its RE (Figure 4B). However, since CPEB1 is also involved in selection of polyadenylation (PA) sites in the 3′UTR of some mRNAs [29], and given the fact that *TWIST1* 3′UTR has three predicted PA sites, we considered the possibility that utilization of a proximal cleavage site could lead to loss of the miR-145-5p RE from the *TWIST1* 3′UTR (Figure 1A). Indeed, cleavage of *TWIST1* 3′UTR at the PA2 site has been predicted to eliminate miR-145-5p RE and thus could be sufficient to prevent miR-145-5p binding. To test this possibility, we used RT-qPCR to measure the accumulation level of *TWIST1* 3′UTR isoforms before and after CPEB1 depletion. We found that partial depletion of CPEB1 had a modest impact (40% increase in the PA3/PA2 ratio) on the utilization of PA3 *versus* PA2 in *TWIST1* 3′UTR (Figure 4C). Our data therefore suggest that CPEB1-directed cleavage/polyadenylation at the PA2 site could also contribute to reducing, albeit to a limited extent, the miR-145-5p effect on *TWIST1* mRNA levels.

### Impact of the TWIST1 /miR-145-5p /CPEB1 pathway on EMT, stemness, and migration

**Figure 5:**
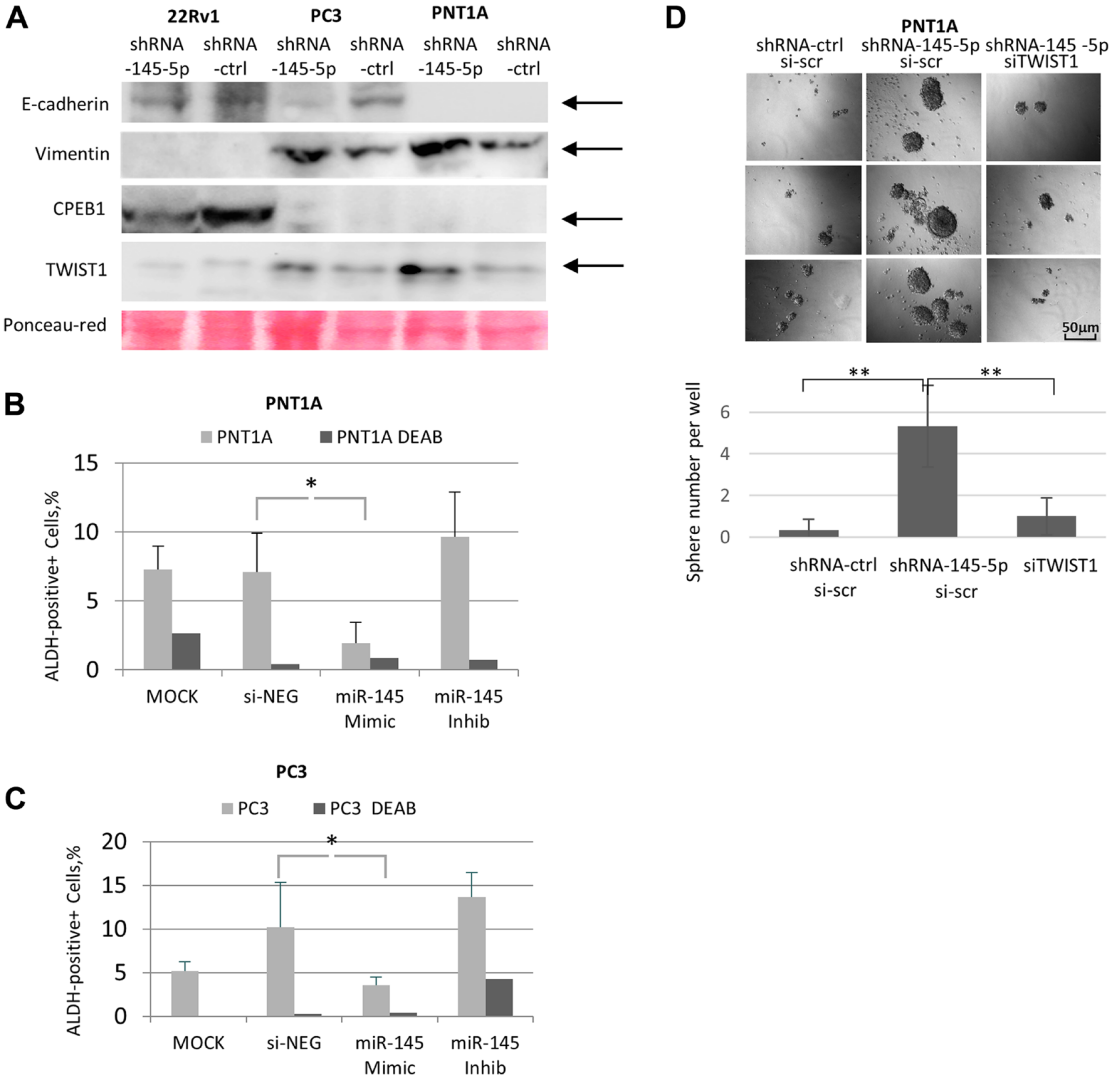
Impact of miR-145-5p on TWIST1, EMT, and self-renewal properties in prostate cell lines. (**A**) ShRNA Mir-145-5p KD up-regulated the mesenchymal marker vimentin together with TWIST1 and down-regulated epithelial marker E-cadherin in PNT1A and PC3 cells (Western blot analysis). This cannot be observed in CPEB1-proficient 22Rv1 cells. (**B** and **C**) MiR-145 mimic transfected into PNT1A and PC3 cells decreased the percentage of ALDEFLORE-positive cells, a stemness marker. Conversely, the miR-145-5p antagomir increased this percentage. ALDEFLOR-positive cells were quantified using the Operetta High-Content Imaging System. (**D**) ShRNA-mediated down-regulation of miR-145-5p increases the number of PNT1A sphere forming units in minimal media, which can be reversed by TWIST1 siRNA silencing. The experiment was carried out in 96-well plates and the sphere number per well was counted. Experiments were repeated 3 times.

It is now well accepted that the EMT is strongly associated with invasive growth of cancer cells and metastasis to distant target organs, as well as with the acquisition of stemness traits implicated in resistance to radio-chemotherapy in cancer patients (1–3). To decipher the biological relevance of the *TWIST1* /miR-145-5p /CPEB1 interplay in the present study, we examined the impact of miR-145-5p depletion on the expression of the two classical EMT markers, E-cadherin (epithelial) and vimentin (mesenchymal), in cells naturally deficient or proficient in CPEB1 (Figure 5A). In CPEB1-limited PC3 and PNT1A cells, miR-145-5p down-regulation led to up-regulation of the mesenchymal marker vimentin and to down-regulation of the epithelial marker E-cadherin, in concert with accumulation of the TWIST1 EMT transcription factor. Conversely, in CPEB1-proficient 22RV1 prostate cells, which exhibit the E-cadherin (+) and vimentin (–) signature according to their epithelial identity, we detected no changes.

It has been previously demonstrated that EMT is often associated with acquisition of stemness traits in cancer cells [2, 3], and that both TWIST1 and miR-145-5p control the acquisition of stem cell properties in these cells [7, 17, 30]. In order to examine these aspects in our experimental setup, we carried out the ALDEFLUOR assay, commonly used for stem cell identification [31, 32]. Our experiments revealed that introduction of the miR-145-5p mimic into PNT1A and PC3 cells down-regulated the number of aldehyde dehydrogenase (ALDH)-positive cells. In contrast, expression of the miR-145-5p antagomir increased the number of ALDH-positive cells (Figure 5B and 5C). These findings highlight the proposed role of miR-145-5p in suppressing the acquisition of stem cell properties. To further confirm this point, we conducted a sphere-forming assay in miR-145-5p-depleted PNT1A cells (Figure 5D) and found that it to be significantly increased after miR-145-5p down-regulation, both in terms of the number of sphere units per well and sphere volume. Furthermore, this phenotype was reversed by TWIST1 siRNA co-silencing (Figure 5D), supporting the notion that the suppressive action of miR-145-5p on the acquisition of stem cell traits is directly linked to its functional interaction with TWIST1 translation.

**Figure 6:**
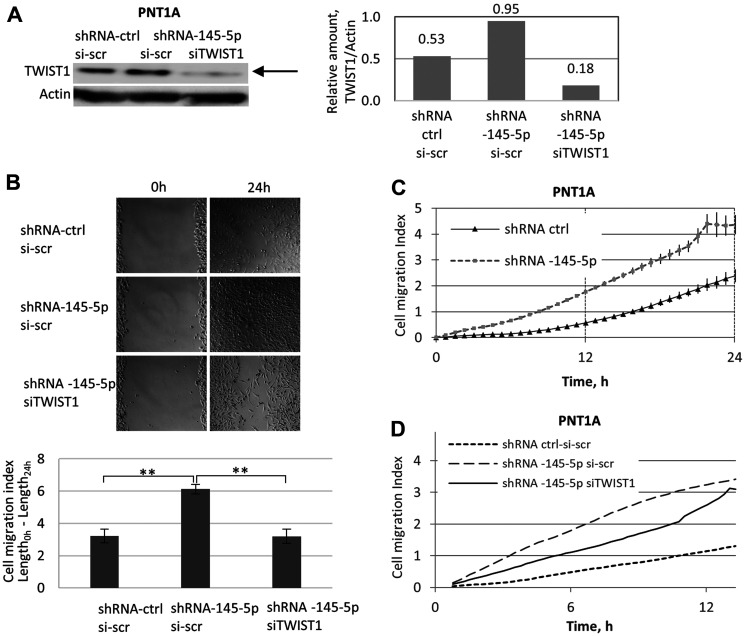
Opposing roles of shRNA-145-5p and TWIST1 siRNA on PNT1A cell migration. (**A**) The impact of shRNA-145-5p and TWIST1 siRNA on TWIST1 expression was evaluated by Western blot analysis. (**B**) Effect of stable expression (miRZip lentivector-based shRNA) alone or co-transfected with TWIST1 siRNA on the migration capacity in PNT1A cells. The cell migration index (wound-healing assay) was quantified in adherent cells by Image J. (**C** and **D**) Effects of time and shRNA-145-5p on the migration of PNT1A cells successively transfected by: i) shRNA-145-5p *versus* shRNA-ctrl (0–24 h post-seeding (C)); and ii) shRNA-145-5p alone or combined with TWIST1 siRNA *versus* the shRNA-ctrl scrambled sequence (0–12 h post-seeding (D)). Experiments were repeated 3 times.

Next, we investigated the impact of shRNA-145-5p and TWIST1 siRNA on the migration potential of PNT1A cells (Figure 6). Along with the expression of the EMT effector vimentin (Figure 5A), depletion of miR-145-5p by shRNA in PNT1A cells (Figure 6A) led to a twofold increase in cell migration capacity measured by the wound-healing method at 24 h post-seeding (Figure 6B). This effect was already visible at 12 h post-seeding according to the exCELLigence technique (Figure 6C). Importantly, this increase in PNT1A cell migration induced by shRNA-145-5p was TWIST1-dependent, since TWIST1 siRNA co-silencing decreased the stimulatory effect of shRNA-145-5p alone on PNT1A migration measured at 6 h and 12 h post-seeding (Figure 6D). Taken together, our data support the implication of miR-145-5p in the mechanisms that control EMT and stemness traits (self-renewal, ALDH positivity) in PNT1A and PC3 cells (Figure 5). We extended these properties to the migratory capacity of PNT1A cells (Figure 6) and confirmed the opposing roles of shRNA-145-5p and TWIST1 siRNA on PNT1A self-renewal and migration (Figures 5D and 6).

### Expression levels of TWIST1, CPEB1, and miR-145-5p in patient-derived PCa cell lines have potential prognosis value

We previously established cancer cell lines from surgically removed clinical prostate tumors. Gene expression analysis in these cell lines revealed transcriptional signatures associated with the clinical outcome, *i. e.,* complete remission or relapse [33]. Then we examined miR-145-5p expression levels by RT-qPCR, and TWIST1 by Western blot in these cell lines.

**Figure 7:**
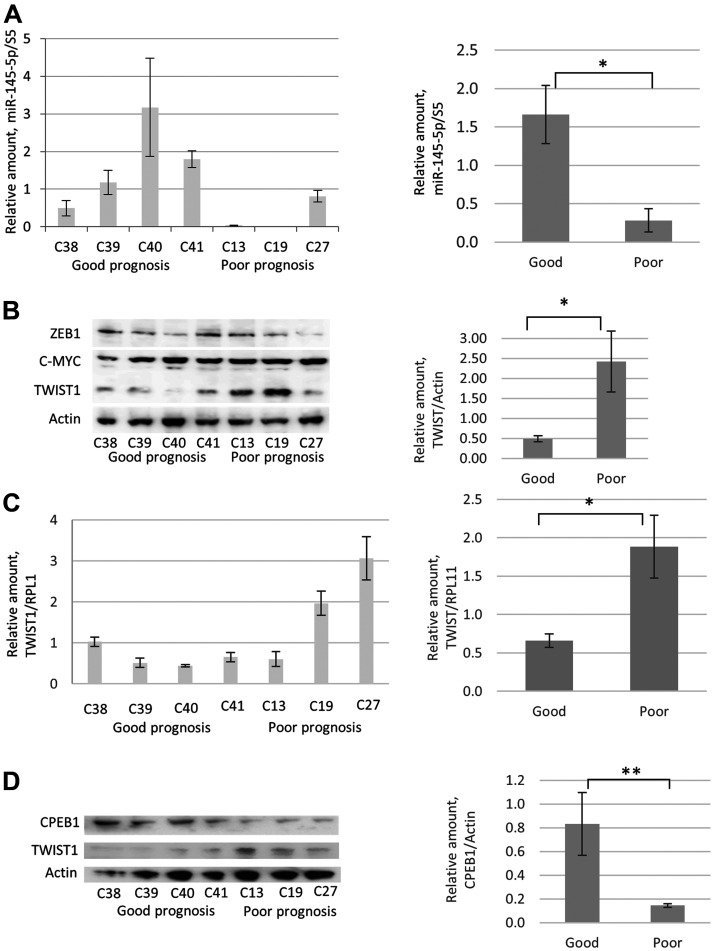
Comparative analyses of miR-145-5p, TWIST1, and CPEB1 expression in prostate cancer cell lines derived from PCa tumor samples from patients with good and poor prognoses. (**A**) RTqPCR analyses revealed low miR-145-5p levels in prostate tumor resections from patients with poor prognoses (C13, C19, and C27) compared with high miR-145-5p levels in prostate tumor resections from patients with good prognoses (C38, C39, C40, and C41). Primary prostate cancer cultures were obtained from freshly explanted prostate cancer specimens subsequently immortalized by transduction of hTERT and SV40 large T antigen, as described [33]. C13, C19, and C27 were established from patients who later relapsed (as defined by the presence of biochemical/local recurrence, metastasis, or disease-specific mortality) and C38, C39, C40, and C41 were established from patients who showed complete remission with surgery alone (10-year follow-up) [33]. Western blot analyses (**B**) and RTqPCR (**C**) show that in tumors of prostate cancer patients, high and low TWIST1 levels respectively correlated with poor and good prognosis. The same correlation is not observed for ZEB1 and c-Myc proteins. (**D**) Western blot analysis reveals that high CPEB1 levels in tumor samples correlate with good prognosis. Experiments were repeated 3 times.

As shown in Figure 7A, cell lines associated with poor prognosis in PCa patients (C13, C19, and C27) showed lower miR-145-5p expression levels by qRT-PCR compared with those established from patients who had a favorable outcome (C38, C39, C40, and C41). In contrast, lower TWIST1 levels were found by Western blot analysis in cell lines established from patients with good prognoses, compared to cell lines obtained from patients who relapsed (Figure 7B and 7C). Interestingly, CPEB1 and TWIST1 protein levels were respectively higher and lower in PCa-derived cell lines associated with good prognosis (Figure 7D). In contrast, no correlation was observed between patients’ prognoses and protein levels of ZEB1 and c-Myc (Figure 7B). Our data indicate that favorable prognoses in PCa patients correlate with high CPEB1/miR-145p and low TWIST1 expression levels in their corresponding PCa-derived cell lines compared to cell lines established from tumor samples collected from patients with relapse.

## DISCUSSION

We have discovered that miR-145-5p mediated control of TWIST1 in human prostate cancer cells is context-dependent, since it relies on the CPEB1 expression level. Our mechanistic studies have revealed so-far unanticipated interplay at the molecular level between miR-145-5p and CPEB1 on TWIST1 translation, thus impacting TWIST1-related phenotype, EMT, self-renewal, and migration. This observation also uncovers the implication of CPEB1 /miR-145-5p interplay in the regulation of TWIST1 expression and cell transformation induced by telomere shortening and CIN accumulation, a hallmark of multistep cancer progression.

We have shown here that miR-145-5p controls TWIST1 expression by targeting *TWIST1* 3′UTR. Stable inhibition of miR-145-5p in immortalized prostate PNT1A epithelial cells and in PC3 cancer cells led to increased TWIST1 expression and consequently to stemness properties (self-renewal in PNT1A and PC3 cells) associated with increased cell migration in PNT1A cells. Strikingly, the function of miR-145-5p as a TWIST1 regulator was masked in 22Rv1 prostate carcinoma cells, which express high levels of CPEB1. In this context, CPEB1 prevents binding of miR-145-5p to RE through binding to the first CPE1 site on *TWIST1* 3′UTR. According to the predicted best energy secondary structure of *TWIST1* 3′UTR, this first CPE1 site is closer to the mir-145-5p RE site, so that CPEB1 could directly interfere with miR-145-5p binding. Alternatively, CPEB1 could also redirect the cleavage/polyadenylation machinery to the second polyadenylation site upstream from the miR-145-5p binding site. Similar interplay between miR-145-5p and CPEB1 interaction in the control of TWIST1 expression was also observed during EMT in the HA5 clone upon CIN+ accumulation, due to telomere shortening [28]. This observation points to the importance of CPEB1/miR-145-5p crosstalk, which drives TWIST1 expression during EMT induced by the accumulation of endogenous DNA damage due to the transition from CIN- to CIN+ in the HEK model [28].

Interestingly, although CPEB1 is able to down-regulate TWIST1 expression by binding to its mRNA, shortening its poly (A) tract, and/or repressing its translation [10, 24], loss of CPEB1 expression is not sufficient to drive TWIST1 re-expression in 22RV1 cells. Thus, TWIST1 expression is increased only when both CPEB1 and miR-145-5p are down-regulated. Considered together, our data support the notion that the tumor suppression activities of miR-145-5p on *TWIST1* translation are regulated by the fine-tuning of CPEB1 expression levels in a context-dependent manner. Accordingly, our data support the potential impact of CPEB1 /miR-145-5p crosstalk on the expression of TWIST1-dependent EMT, and perhaps on its reversion (MET). Both EMT and MET are associated with cellular plasticity during embryonic and early post-natal development, tissue repair, and cancer progression. In this context, it should be considered that partial forms of EMT could be sufficient to drive invasiveness by disrupting E-cadherin-mediated cell-cell adhesion and promoting invadopodia formation in cancer cells [34].

TWIST1 is a major driver of EMT, and EMT is often associated with metastasis, stemness, and drug resistance in many cancers, including PCa [1, 3]. Therefore, regulation of TWIST1 expression *in vivo* could be a key point in progression of prostate cancer toward the metastatic stage. On the other hand, miR-145-5p down-regulation is often associated with advanced stage PCa [11–16], metastasis [17], and has also been reported to be involved in control of stem cell traits [20]. Our data suggest that miR-145-5p is a major factor in the control of TWIST1 expression in prostate cancer, and that loss of miR-145-5p may constitute a key step in progression toward metastasis. Importantly, our data also suggest that loss of CPEB1 expression may be required to allow cancer cells to proceed to a metastatic stage with the acquisition of stem cell traits. Finally, the fact that CPEB1 and miR-145-5p were both down-regulated, and that TWIST1 was up-regulated in PCa cell lines derived from the tumors of patients who relapsed, suggests that determining such markers in clinical samples may bear prognostic value, along with Gleason scores, tumor staging, and PSA levels.

To conclude, our work provides evidence that miR-145-5p and CPEB1 functionally overlap to control TWIST1 expression in prostate cancer cells by preventing EMT expression, cell migration, and stem cell properties. These observations have important clinical implications, since they may provide a better definition of prognostic markers and of potential drug targets in prostate cancer patients.

## MATERIALS AND METHODS

### Plasmid constructs, cell lines, transfection and transduction

The *TWIST1* 3′UTR sequence (nt 926-1634, [GeneBank: NM_011658]) without the intron was cloned into XhoI/NotI sites in psiCheck2 (wt pA3) (Promega) [10].

The PNT1A cell line was derived from normal post-pubertal human prostate epithelial cells transformed with SV40 [35]. The PC3 cell line was established from bone metastases of grade-IV prostate cancer [36]. 22Rv1 is a human prostate carcinoma epithelial cell line derived from a xenograft that was serially propagated in mice after castration-induced regression and relapse of the parental androgen-dependent CWR22 xenograft [37]. Both PC3 and 22Rv1 are highly tumorigenic in nude mice, whereas PNTA1 is not. All prostate cell lines were cultured in RPMI media (ThermoFisher) with 10% FBS.

Primary prostate cancer cultures were obtained from clinical prostate cancer specimens and immortalized by transduction of hTERT and SV40 large T antigen, as described [33]. These PCa cell lines obtained from prostate cancer patients were cultured in IMDM media (ThermoFisher) with 10%FBS.

HEK cells were established by transfecting primary human embryonic epithelial kidney cells, using a plasmid carrying ER-SV40 and a neo-resistance cassette (Silvia Bacchetti, McMaster University, Hamilton, Canada), as described [38]. Immortalized HEK cells were derived from the HA5 clone, as previously described [28]. HEK cells (HA5 Early and Late passages), PNT1A, 22Rv1 and PC3 cells stably expressing miR-145-5p antagomir (miRZIP-145, System Biosciences) were established as follows: 2 μg of the pGreenPuro lentivector (MZIP145-PA-1-GVO-SBI) expressing the shRNA targeting miR-145-5p or scrambled sequence were transfected to 293 cells, using lipofectamine 2000 (ThermoFisher). Viral particles were collected and used to infect HA5, PNT1A, PC3, and 22Rv1 cells. Twenty-four hours after addition of viruses, transduced cells were selected by adding 0.5 μg/ml puromycin to the growth media for one week. TWIST1 siRNA knock-down (KD) was carried out as follows: PNT1A cells stably induced with MiRZIP-145-5p were transfected with TWIST1 siRNA (4204795-F, Sigma) and scrambled siRNA (siRNAscr-1 529856, Sigma) in RPMI media, using lipofectamine RNAiMax transfection reagent (ThermoFisher). TWIST1 silencing was confirmed 72 h post-transfection by Western blot. siCPEB1 KD was performed with CPEB1 siRNA (siCPEB1 - HA05434622, Sigma), as was described for siTWIST1 KD.

### Dual luciferase reporter assay

All transfections were carried out in triplicate in 96-well plates, using 200 ng of DNA and lipofectamine 2000 (ThermoFisher). MiR-145-5p mimic-(MC11480, Ambion) and anti-miR (MH11480, Ambion) were used at 50 nM concentration. Cells were lysed after 20 h of transfection, using Passive Lysis Buffer (Promega). Firefly and Renilla luciferase activities were measured by FLUOstar OPTIMA (SMG LABTECH, Promega). Results are expressed as the ratio of Renilla to Firefly values.

### Total RNA extraction, reverse transcription (RT) and quantitative (q) PCR

Total RNA was purified using TRIzol (ThermoFisher), according to the manufacturer’s instructions. Total RNA (250–500 ng) was used for oligo-dT (or random) primed reverse transcription by SuperScript III reverse transcriptase (ThermoFisher). The resulting cDNA was quantified by qPCR, using SybrGreen GoTaq Mastermix (Promega) and the Roche LC480 instrument. For each sample, qPCR reactions were done in triplicates. Fold change was calculated relatively to ACTB (housekeeping) using the 2−ΔCT method [39].

### QPCR primers

B-Actin-F CACCATTGGCAATGAGCGGTTC

B-Actin-R AGGTCTTTGCGGATGTCCACGT

GAPDH-F CTGAGCAGACCGGTGTCACATC

GAPDH-R GAGGACTTTGGGAACGACTGAG

TWIST1-F GCCAGGTACATCGACTTCCTCT

TWIST1-R TCCATCCTCCAGACCGAGAAGG

Mature miR-145-5p was quantified using the miRCURY system (EXIQON) with 250 ng of DNase-treated total RNA in three independent RT reactions, and qPCR, using miR-specific locked nucleic acid primers (204483, EXICON). The mean expression of U6 (203907, EXICON), or 5S (203906, EXICON) was used as an endogenous control for relative quantification according to 2−ΔCT method.

### RNA pull-down by biotinylated miRNA

MiR-145 mimic (MC11480, Ambion) was biotinylated with the 3′-end Biotinylated Kit (Pierce) and transfected to the PNT1A and PC3 cells with the Lipofectamine RNAiMAX reagent (ThermoFisher). Biotinylated RNA supplied with the kit was used as the control. Biotin pull-down was performed as follows: 11 × 10^6^ cells were centrifuged 48 h after transfection at 1000 rpm for 2 min. Pellets were incubated for 15 min on ice in Pierce lysis buffer (25 mM Tris-HCl pH 7.4, 150 mM NaCl, 1 mM EDTA, 1% NP-40, and 5% glycerol) with protease and RNAs inhibitors, then centrifuged (15 min, 13200 rpm). The supernatant was collected and supplemented by an equal volume of 2× TNT buffer (50 mM Tris-Cl, pH 8.0, 2 mM EDTA, and 150 mM NaCl, 1% Triton X-100). Streptavidin agarose beads (30 μl per reaction) were washed 3 times in TENT buffer (2 min, 2000 rpm at 4°C), incubated 30 min at 20 rpm at RT with rotation. Beads were washed 3 times in 500 μl PBS. RNA was purified with TRIAZOL regent and used for qPCR reaction with specific primers:

### Wound healing closure and migration assays

Cells were plated and grown to confluence in 6-well plates. Pipette tips were used to create streaks in the monolayers. Wound-healing was analyzed and quantified, using the Image J program.

Cell migration experiments were carried out using the exCELLigence RTCA-DP system (Roche) as previously described [40]. Briefly, cells were serum-starved for 6 h prior to the assay. First, 160 μl of RPMI 1640 supplemented with 5% FBS was added to the lower chamber of CIM-Plate 16, and 30 μl of serum-free medium (SFM) was added to the upper chamber. Second, 4 × 10^4^ cells per well were re-suspended in 100 μl of SFM and loaded into the upper chamber. Third, the 16-well CIM-plate containing the cells was plated onto the RTCA DP Analyzer inside the incubator at 37°C for measurement at 15-min intervals, and cells that migrated to the lower chamber were monitored. Kinetic information concerning the migration of the cells was recorded using real-time cell analysis and the cell index slope (1/h) for a time period/range calculated according to the RTCA Software Manual (Version 1.2).

### Self-renewal spheroid formation assay

In total, 500 cells per well were seeded into a 96-well Ultra Low Cluster plate (174927 96F Bottom Plate, Thermo Scientific) and cultured in suspension in serum-free DMEM-F12 medium supplemented with 3mM glutamine, 0.6% glucose, 4 μg/ml insulin (Sigma-Aldrich), 20 ng/ml hEGF (R&D Systems), 10 ng/ml hBasic-FGF (R&D Systems), and B27 supplement. After 10–12 days, the number of spheres (tight, spherical, non-adherent masses above 50 μm in diameter) were counted, using an inverted microscope (Axiovert 135, Zeiss Germany).

### Protein extraction and immunoblotting

Protein extracts were prepared by scraping the cells in the lysis buffer containing 100 mM KCl, 5 mM MgCl2, 20 mM Hepes-KOH (pH 7.8), 2 mM DTT, 25% NP-40, and a protease inhibitor cocktail (Roche). 20 μg of the whole protein extract was loaded onto 4–12% NuPAGE Bis-Tris Pre-Cast Gels and resolved using the NuPAGE System followed by iBlot transfer (ThermoFisher). Primary antibodies were: CPEB1 dilution 1:1000 (ab73287 Abcam) TWIST1 H-81, dilution 1:1000 (sc-15393, Santa Cruz Biotechnology), anti-c-Myc 9E10, dilution 1:1000 (11 667 149 001 ROCHE), anti-ZEB1 OTI3G6, dilution 1:1000 (TA802298 Origene), β-Actin (C4) HRP, dilution 1:2000 (sc-4778 HRP Santa Cruz Biotechnology).

### Statistical analyses

Data are means +/− SD of at least three separate experiments. Statistical significance was assessed with Student’s *t*-test. ^*^ *p* < 0.05; ^**^ *p* < 0.01.

## Abbreviations

EMT: Epithelial-to-Mesenchymal Transition
shRNA: short hairpin RNA
miRNA: microRNA
CPEB1: Cytoplasmic Polyadenylation Element Binding protein1
PCa: Prostate Cancer
SHH: Sonic HedgeHog
FGF: Fibroblast Growth Factor
BMP: Bone Morphogenetic Protein
HEF1: Human Enhancer of Filamentation1
3′UTR: 3′Untraslated Region
ALDH: Aldehyde dehydrogenases
R-luc: Renilla-luciferase
FF-luc: Firefly-luciferase
Ctrl: control
